# EMILIN-1 in the tumor microenvironment: insights from CNS tumors and beyond

**DOI:** 10.3389/fonc.2026.1771697

**Published:** 2026-04-01

**Authors:** Kirill A. Arsentiev, Abigail Fajardo, Arseniy E. Yuzhalin

**Affiliations:** 1 Research Center for Translational Medicine, Sirius University of Science and Technology, Sochi, Russia; 2 The University of Texas Health Science Center, Houston, TX, United States

**Keywords:** cancer, CNS tumors, EMILIN-1, extracellular matrix, glioblastoma multiforme, tumor microenvironment, tumor, vasculature

## Abstract

Elastin Microfibril Interface Located Protein 1 (EMILIN-1) is a multifunctional extracellular glycoprotein, primarily involved in maintaining tissue homeostasis. Known for regulation of elastogenesis and vascular stability, EMILIN-1 has emerged as a key modulator of multiple biological processes, including cell adhesion, migration, and proliferation. Recent experimental data highlight the importance of EMILIN-1 in cancer biology, especially in malignant tumors of the nervous system, where EMILIN-1’s regulatory functions influence tumor progression and metastatic potential. Specifically, EMILIN-1 can exert tumor-suppressive effects by modulating cell signaling pathways of tumor cells and EMILIN-1 can also alter the immune response and promote progression of brain tumors. This review article provides a comprehensive analysis of the recently discovered mechanisms through which EMILIN-1 promotes tumor progression in the nervous system and other sites.

## 1 Introduction

The tumor microenvironment (TME) is a dynamic ecosystem defined by the complex interplay between cancer cells, various non-cancerous stromal populations, and a specialized acellular scaffold known as the extracellular matrix (ECM) (1). Within this niche, tumor growth, invasion, and metastasis are driven by the reciprocal communication between these cellular components, mediated through both direct cell-cell interactions (2, 3) and the surrounding ECM (4). This multi-faceted interaction is further modulated by physiological constraints, including availability of oxygen (5) and essential nutrients (6), and the activity of diverse secreted molecules (proteins, DNA and RNA) (7).

Far from being a passive structural scaffold, the ECM is now recognized as an active and instructive regulator of tumor progression. In the specific context of the central nervous system (CNS), brain tumors such as gliomas actively orchestrate a self-advantageous extracellular matrix to promote recurrence, aggressive invasion, and resistance to therapy (8). This “matrix orchestration” involves the continuous biochemical and biophysical remodeling of a complex network of macromolecules (9). Consisting of secreted glycoproteins, proteoglycans, collagens, and non-protein elements like hyaluronic acid, the ECM serves as a pivotal regulatory hub. It integrates mechanical support with the presentation of cell signaling molecules, ultimately modulating cell proliferation (10), angiogenesis (9), and the suppression of local immune responses (11, 12). While cancer-associated fibroblasts (CAFs) (13) are the primary contributors to the production of these matrix proteins in systemic cancers, in the CNS, other cell types, including microglia, astrocytes, and the cancer cells themselves (14), actively participate in depositing and modifying the unique brain ECM.

EMILIN-1 (Elastin Microfibril Interface Located Protein 1) is an ECM glycoprotein primarily involved in maintaining tissue elasticity and structural integrity, particularly within the vascular system. Functionally, EMILIN-1 has been primarily associated with influencing arterial pressure (15), cell adhesion and migration (16), cell proliferation (17), angiogenesis (18), and lymphangiogenesis (19). In recent years, multiple groups explored the potential implications of EMILIN-1 and other proteins belonging to the EMILIN/Multimerin family in the CNS development. Furthermore, accumulating evidence indicates that EMILIN-1 is involved in critical stages of tumor development, including tumor initiation, growth, invasion, and metastasis (20, 21). The function of EMILIN-1 in the development of primary CNS tumors, peripheral nervous system tumors, and CNS metastases is a subject of particular interest, in view of recent studies that have demonstrated a distinctive mechanism for EMILIN-1-regulated suppression of the immune surveillance (20). While CAFs are major ECM producers in many solid tumors, the cellular source of EMILIN-1 varies by tumor type. In brain metastasis, tumor-educated astrocytes are the primary source (20). In primary brain tumors, the cellular origin remains incompletely characterized but likely involves vascular cells, reactive astrocytes, and tumor cells themselves, depending on tumor type.

Recent reviews have comprehensively covered the EMILIN family structure and general functions (22, 23). Expanding on these works, the present review provides a distinct focus by (1): systematically examining EMILIN-1’s context-dependent and often contradictory roles specifically in brain tumors and brain metastasis, (2) critically evaluating the mechanistic evidence (or lack thereof) underlying reported associations, (3) integrating recent transcriptomic and proteomic data from human brain tissues, and (4) proposing a conceptual framework to reconcile opposing findings across tumor types. This article aims to summarize the biology of EMILIN-1 and review its role in tumor development, with a particular emphasis on primary and secondary malignancies of the CNS.

## 2 Gene and protein structure of EMILIN-1

EMILIN-1 was originally identified in chick blood vessels as glycoprotein 115 (24) and is now recognized as an elastic fiber ECM glycoprotein of the gC1q/TNF superfamily and part of the EMILIN/Multimerin family (25). Its gene is located on chromosome 2 in mammals, and point mutations have been linked to hypertension, neuropathy, and connective tissue disease, highlighting its role in tissue homeostasis (19, 25–28, 73). In normal tissues, EMILIN-1 is primarily produced by vascular smooth muscle cells, blood endothelial cells, and lymphatic endothelial cells (19, 26).

Structurally, mature EMILIN-1 (104.5 kDa) comprises an N-terminal cysteine-rich EMI domain, a gC1q domain, a collagenous (COL) domain, a coiled-coil region, and two leucine zippers (29). The EMI domain mediates multimerization via interactions with EMI domains of other proteins, including pro-TGF-β1 (30), and also facilitates EMILIN-1 binding to α4β1 and α9β1 integrins, which is critical for its anti-proliferative effects in cancer cells (16, 17). The coiled-coil region enables oligomerization and assembly into higher-order structures (29). The gC1q domain enables supramolecular assembly through disulfide bonds (25) and, importantly, contains an integrin-binding motif (RGD) that mediates α4β1 and α9β1 integrin-dependent cell adhesion (30–32). Additionally, this domain sequesters latent TGF-β in the extracellular matrix, preventing its activation and thereby inhibiting TGF-β-dependent tumor-promoting signaling (15, 33).

Functionally, EMILIN-1 acts as a negative regulator of TGF-β signaling by retaining latent TGF-β in the ECM and blocking its bioavailability (15, 33). This inhibitory function is particularly relevant in cancer, where TGF-β can drive epithelial-mesenchymal transition, immune suppression, and metastasis (33, 34). Together, the integrin-binding and TGF-β-regulatory domains position EMILIN-1 as a key ECM component capable of directly modulating tumor cell behavior and the tumor microenvironment.

## 3 Physiological functions of EMILIN-1

### 3.1 Vascular system

In normal tissues, EMILIN-1 is produced by vascular smooth muscle cells, blood endothelial cells, and lymphatic endothelial cells, where it contributes to blood vessel integrity and elasticity (19, 24, 26, 32). EMILIN-1 deficiency in mouse models leads to reduced mesenteric artery diameter and hypertension, as well as aberrant aortic valve angiogenesis, interstitial cell activation, and neovascularization (15, 18). These vascular abnormalities are attributed to EMILIN-1’s role as a negative regulator of TGF-β signaling. In particular, its EMI domain binds pro-TGF-β, preventing furin-mediated cleavage and thereby reducing mature TGF-β bioavailability (15, 33, 35). TGF-β is a known promoter of vascular remodeling, and its dysregulation contributes to pathological angiogenesis.

EMILIN-1 also critically regulates lymphatic vasculature. *Emilin-1*^-^/^-^ mice exhibit lymphatic hyperplasia, a 67% increase in proliferating LECs (vs. 24% in wild-type), vessel dilation, dysmorphic network formation, impaired lymph drainage, enhanced lymph leakage, and mild lymphedema (21). EMILIN-1 is also a structural component of lymphatic collector valves, where it contributes to valve integrity and function (19). These phenotypes demonstrate that Emilin-1 suppresses excessive LEC proliferation and maintains normal lymphatic structure and function. Unlike developmental lymphatic genes such as VEGF-C, PROX-1, and FOXC-2, EMILIN-1 is not required for lymphatic specification but rather restrains pathological lymphangiogenesis (36).

Both vascular and lymphatic phenotypes arise from loss of EMILIN-1-mediated TGF-β inhibition. Notably, these findings are derived primarily from *Emilin-1*^-^/^-^ mouse models. Validation in human tissues through immunohistochemical analysis of tumor vasculature and correlation with clinical outcomes remains limited (21). Nevertheless, the consistent suppressive effects of EMILIN-1 on endothelial cell proliferation and aberrant vessel formation position it as a potential endogenous inhibitor of tumor-associated angiogenesis and lymphangiogenesis. In the tumor microenvironment, where TGF-β is often overactive and promotes immune suppression, EMT, and metastasis, EMILIN-1 downregulation may represent a permissive switch for vascular remodeling that facilitates tumor progression (33, 34).

### 3.2 Connective tissue

EMILIN-1 regulates connective tissue architecture, and its aberrant function can lead to structural defects (37). In 2015, the first disease-causing *EMILIN-1* mutation was identified: a heterozygous c.64G>A (p.A22T) substitution segregating over three generations in a family with an autosomal dominant connective tissue disorder resembling Marfan syndrome (MFS), including aortic aneurysms, peripheral neuropathy, arthropathy, and increased skin elasticity (37). Despite MFS-like symptoms, patients lacked Fibrillin-1 deficiency. The p.A22T mutation prevents proper protein cleavage, causing EMILIN-1 accumulation in the ER and reduced ECM deposition (37).

Subsequent studies clarified EMILIN-1 assembly mechanisms. Contrary to earlier reports suggesting Fibrillin-1 dependence (38), EMILIN-1 was found to colocalize with Fibrillin-2 rather than Fibrillin-1, consistent with the p.A22T phenotype (39). In MC3T3-E1 pre-osteoblasts and primary calvarial osteoblasts, EMILIN-1 assembly required fibronectin but not fibrillins (39). *Emilin-1* knockout disrupted Fibulin-4 deposition, while *Fibulin-4* knockdown did not affect Emilin-1, positioning Emilin-1 upstream of Fibulin-4 in ECM organization. Both proteins are colocalized in murine calvarial bone (39). These findings implicate EMILIN-1 not only in connective tissue maintenance but also potentially in bone matrix regulation.

### 3.3 Neural tissue

**Figure 1:**
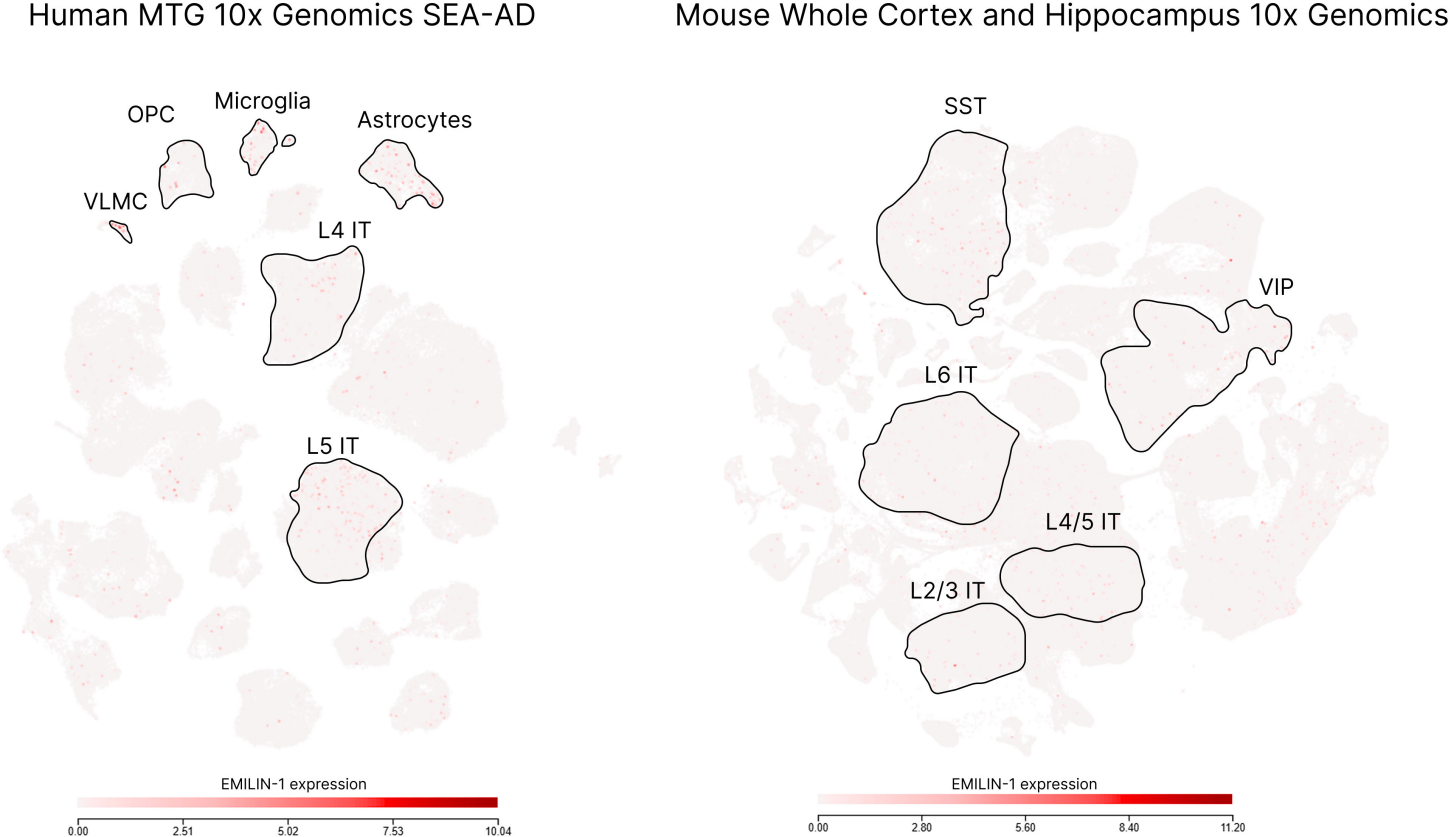
Visualization of single-cell RNA-sequencing data from human and mouse brain regions, colored by EMILIN-1 expression levels (Allen Brain Atlas datasets). MTG, middle temporal gyrus; OPC, oligodendrocyte progenitor cells; VLMC, vascular leptomeningeal cells; L4 IT, layer 4 intratelencephalic-projecting glutamatergic neurons; L5 IT, layer 5 intratelencephalic-projecting glutamatergic neuron; SST, somatostatin-expressing inhibitory interneurons; VIP, vasoactive intestinal polypeptide-expressing inhibitory interneurons.

Although studies on the role of EMILIN-1 in the nervous system are sparse, evidence indicates its significance in both physiological and pathophysiological conditions. Generally, its expression in mammalian neural cells under normal conditions is low or nearly absent. According to the Allen Brain Atlas data (https://portal.brain-map.org/atlases-and-data/rnaseq), the expression of EMILIN-1 in the human brain is most prominent in astrocytes, microglia, oligodendrocyte progenitor cells (OPCs), vascular leptomeningeal cells (VLMCs), and glutamatergic cortical neurons. In mouse brain regions, Emilin-1-positive cells are more abundant in clusters of excitatory cortical neurons and specific subpopulations (e.g. VIP+ cells, SST+ cells) of GABAergic neurons (**Figure 1**). These data point to possible involvement of EMILIN-1 in both neuronal and glial functions.

EMILIN-1 protein was localized in the perineurium and epineurium of human motor nerves, where it was co-localized with neuroectodermal marker S100b (40). Importantly, a missense mutation c.748C>T [p.R250C] in the *EMILIN-1* gene has been identified as a causative factor in the development of distal motor neuropathy. The knockout of emilin1a (an orthologue of the human gene) in zebrafish (Danio rerio) resulted in developmental delay, locomotion defects, and abnormal growth of motor neuron axons in the spinal cord (40). As demonstrated in previous studies (41), zebrafish have also exhibited evidence for the expression of *Emilin-1* in hindbrain and midbrain, thus indicating the potential involvement of this protein in the CNS development.

In view of the established role of EMILIN-1 in angiogenesis and TGF-β signaling (see 3.1.), a number of other potential functions in the central nervous system can be postulated, although these remain to be definitively demonstrated. It is evident that TGF-β signaling plays a crucial role in the development of the nervous system (42). As a multifunctional ECM protein, EMILIN-1 has the capacity to regulate a variety of processes, including axonal guidance, cell migration and survival, as well as the inflammatory responses of glial cells. In consideration of the established function of EMILIN-1 in vascular development and blood pressure regulation, it is plausible that EMILIN-1 contributes to the development of brain vascularity and maintenance of blood-brain barrier homeostasis. For instance, dysregulation of EMILIN-2, another protein belonging to the EMILIN/Multimerins family, has been demonstrated to contribute to the development of brain arteriovenous malformations (AMV) (43).

Higher expression in the CNS has been demonstrated for other EMILIN/Multimerin family proteins. For example, *Emilin-3* is transiently expressed from E10.5 to E12.5 in the midbrain of mice, while *Emilin-2* is expressed in the neural tube (44, 45). However, the specific functions of these proteins in the nervous system remain to be determined. In this regard, the question of the role of EMILIN proteins, especially EMILIN-1, in normal physiology and pathology requires further research.

## 4 EMILIN-1 in primary tumors of the nervous system

**Figure 2:**
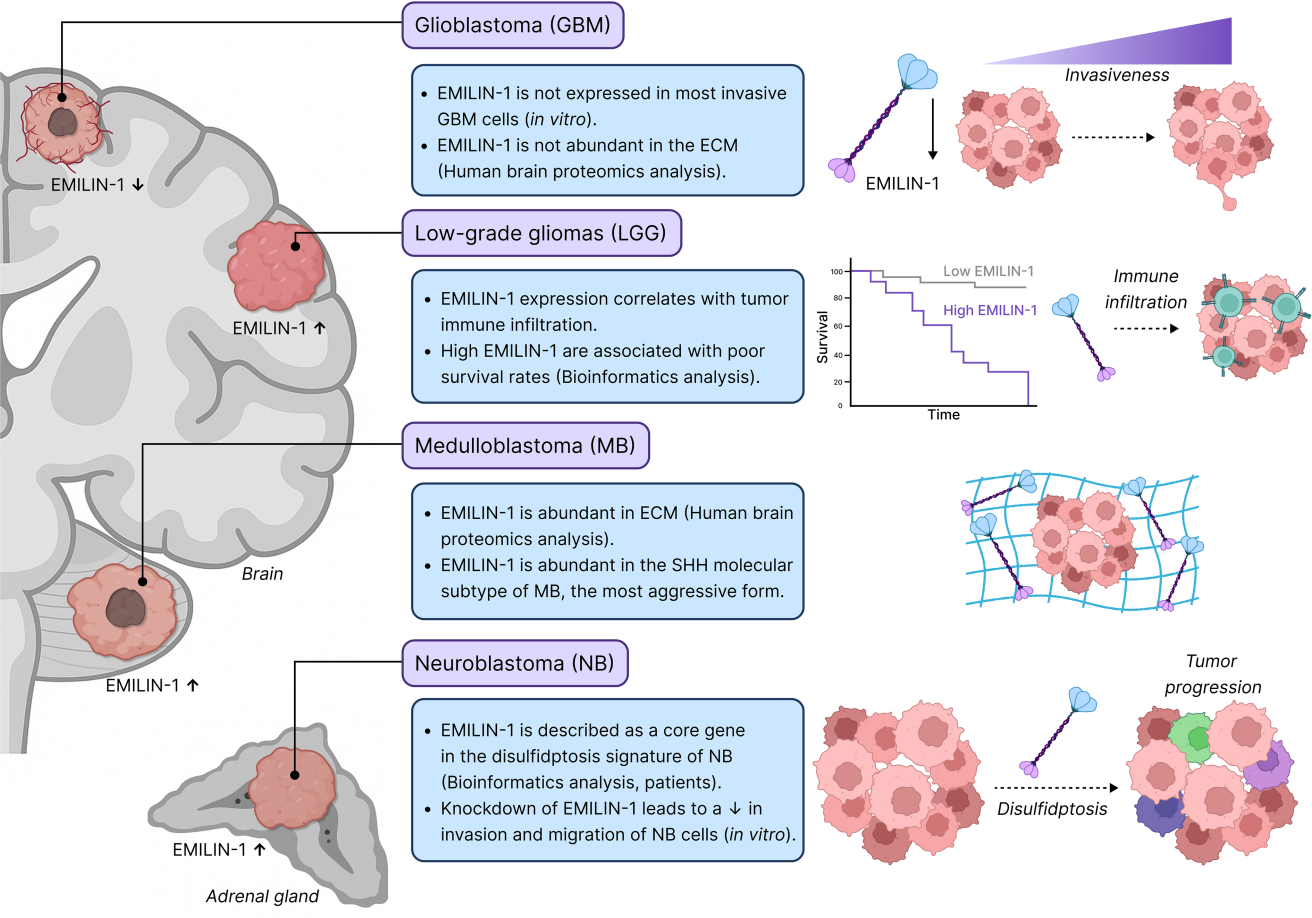
Context-dependent roles of EMILIN-1 in primary brain tumors and neuroblastoma. In glioblastoma (GBM), EMILIN-1 downregulation correlates with increased invasiveness, though proteomic data show no significant EMILIN-1 upregulation in human tumors ECM. Conversely, in medulloblastoma (MB), EMILIN-1 is elevated in the ECM. In low-grade gliomas (LGG), EMILIN-1 level correlates with immune infiltration and is associated with poorer patient survival. In neuroblastoma (NB), EMILIN-1 is described as a “core” gene in disulfidptosis signature, while its knockdown reduces invasion/migration/colony formation *in vitro*. Solid lines indicate established mechanisms; dashed lines indicate proposed or correlative relationships. Arrows denote associations or correlations rather than proven causal relationships unless specifically validated by functional studies.

Recent research demonstrated an involvement of EMILIN-1 in the development of primary CNS tumors, yet these studies led to apparently conflicting results (**Figure 2**). In tumors, EMILIN-1 can be produced by tumor cells themselves, CAFs, endothelial cells, and astrocytes, with the predominant cellular source varying by tumor type and potentially by disease stage.

### 4.1 Glioblastoma and medulloblastoma

Mass spectrometry (MS)-based proteomics analysis of ECM of human glioblastoma (GBM) and medulloblastoma (MB) tissue explants showed increased abundance of EMILIN-1 only in the microenvironment of MB, but not in GBM (46). While the abundance of EMILIN-1 was higher in GBM compared to normal isocortex, this difference was not statistically significant. Interestingly, in MB patients EMILIN-1 was mostly overexpressed in subjects with the SHH molecular subtype characterized by Sonic Hedgehog pathway activation, worst prognosis, and enrichment for MYCN amplifications (46). In another study, an *in vitro* analysis of the secretome of several glioblastoma cell lines (LN18, T98, U118 and U87) demonstrated an inverse correlation between EMILIN-1 expression and the invasiveness of the cell line. Whereas EMILIN-1 was overexpressed in the less invasive cell lines LN18 and U118, the highly invasive U87 GBM cell line did not secrete any EMILIN-1 in the conditioned medium (47). These findings suggest a possible role for EMILIN-1 in regulating GBM infiltration, which could be consistent with tumor-suppressive activity, in contrast to the phenotype observed in MB. The mechanisms underlying differential EMILIN-1 expression between GBM and MB may involve tumor-specific transcriptional regulation, differences in stromal cell composition, or distinct patterns of ECM remodeling. The absence of EMILIN-1 in GBM ECM despite its presence in MB suggests active suppression or degradation mechanisms in GBM.

### 4.2 Low-grade glioma

Zhao et al. (2020) conducted a bioinformatics analysis to identify the prognostic value of EMILIN/Multimerin family gene expression in tumors of patients with low-grade gliomas (LGG). In contrast to GBM, LGG was characterized by the elevated expression of EMILINs/Multimerins, including EMILIN-1. Higher expression of EMILIN/Multimerins served as a significant predictor of adverse clinical outcomes, specifically a low probability of recurrence-free survival, in individuals diagnosed with LGG (48). They also reported correlations between elevated *EMILIN-1* expression and increased infiltration of various immune cell types in LGG, including B cells, CD4+ T cells, neutrophils, macrophages, and dendritic cells. While these correlational data suggest EMILIN-1 may influence immune cell recruitment, direct mechanistic evidence demonstrating that EMILIN-1 drives immune infiltration remains to be established through functional validation studies (48). Interestingly, while no significant correlation emerged between *EMILIN-1* expression and CD8+ T-cell infiltration in LGG (48), a distinct pattern has been observed in other malignancies. In breast cancer, for instance, CD8+ T cells were found to preferentially localize within EMILIN-rich regions, suggesting tumor type-specific differences in ECM-mediated immune cell recruitment (49).

### 4.3 Neuroblastoma

Recent studies have uncovered, linking *EMILIN-1* with disulfidptosis, a recently identified form of regulated cell death mediated by excessive cystine uptake leading to cytoplasmic NADPH depletion and disulfide accumulation, ultimately triggering the formation of abnormal disulfide bonds in actin cytoskeleton proteins and disruption of the actin filament network, ultimately resulting in cell death (50). This variant of programmed cell death was shown to influence the initiation and progression of various tumors (51). For gliomas, a higher degree of disulfidptosis was associated with poor overall survival, suggesting that high expression of disulfidptosis-related genes may serve as a potential biomarker for glioma malignancy. Disulfidptosis may be associated with increased adaptability of tumor cells to adverse survival conditions, promoting tumor cell proliferation and invasion under unfavorable microenvironmental conditions. This has been documented for gliomas of varying degrees of severity (52). Mengzhen et al. (2024) utilized machine learning to analyze potential therapeutic targets in the context of disulfidptosis in neuroblastoma (NB), identifying *EMILIN-1* as a “core gene” in a 44-gene signature controlling disulfidptosis in human NB clinical specimens and cell lines SKNAS and SH-SY5Y. Specifically, it was shown that *EMILIN-1* along with another core gene *CYFIP1* were actively expressed in NB tissues, while knockdown of these two genes affected multiple tumor phenotypes in NB cells, including colony formation, migration and invasion. In addition, an elevated expression of the *EMILIN-1* was associated with unfavorable prognostic effects for NB patients (53).

However, these conclusions rely primarily on bioinformatic and knockout studies, without direct mechanistic evidence linking extracellular EMILIN-1 to intracellular disulfide stress or NADPH depletion. A potential mechanism may involve integrin-mediated signaling, as EMILIN-1 is a secreted ECM glycoprotein that interacts with various integrins. Integrin activation can facilitate the recruitment and activation of the Rac1-WAVE regulatory complex (WRC), which includes the other “core gene” *CYFIP1*. The Rac1-WRC pathway has been recently shown to play a critical role in the formation of aberrant disulfide bonds and subsequent actin cytoskeleton collapse characteristic of disulfidptosis (54). Thus, the EMILIN-1-integrin signaling axis represents a plausible mechanistic bridge between the extracellular environment and the intracellular metabolic stress that triggers disulfidptosis in NB.

### 4.4 Context-dependent mechanisms underlying EMILIN-1’s dual roles

Based on correlative analyses, *EMILIN-1* expression appears to be associated with immune microenvironment characteristics in LGG and with disulfidptosis-related signatures in NB. The precise mechanisms by which extracellular EMILIN-1 might influence these processes require experimental validation, including functional studies that directly test EMILIN-1’s role in immune cell recruitment and disulfidptosis induction.

The contradictory roles of EMILIN-1 across different nervous system tumors (i.e. tumor-suppressive in GBM, tumor-promoting in LGG, and associated with cell death pathways in NB) demand a unifying conceptual framework. We propose that EMILIN-1’s functional outcomes may depend on multiple context-dependent factors.

First, different cell types produce EMILIN-1 in different tumor contexts. In GBM, reduced EMILIN-1 may reflect loss of vascular smooth muscle cell or pericyte populations after treatment (55). In brain metastasis (as discussed below), astrocytes are the primary source. The cellular origin likely determines EMILIN-1’s spatial distribution, local concentration, and interaction partners.

Second, a full-length EMILIN-1 may have tumor-suppressive functions through TGF-β sequestration in the ECM and resulting integrin-mediated anti-proliferative signaling. However, proteolytically cleaved EMILIN-1 fragments, generated by matrix metalloproteinases or neutrophil elastase (16, 56), may lose tumor-suppressive activity or even acquire pro-tumorigenic functions. The balance between full-length and cleaved forms likely varies by tumor type and inflammatory status.

Third, EMILIN-1’s effects are mediated through α4β1 and α9β1 integrins (16, 17). Differential expression of these integrins across tumor types, or their engagement by competing ECM ligands, could alter EMILIN-1’s functional outcomes. In contexts where integrin signaling promotes cell survival (e.g., through FAK/PI3K/AKT pathways), EMILIN-1-integrin interactions might paradoxically support tumor growth. Conversely, in contexts where integrin engagement triggers anti-proliferative signals, EMILIN-1 would act as a tumor suppressor.

Fourth, the composition of the surrounding ECM, the activity of TGF-β and other growth factors, the degree (intensity) and phenotype of immune cell infiltration, and the degree of hypoxia all influence EMILIN-1’s effects. For example, in highly inflamed tumors with abundant neutrophils, EMILIN-1 may be rapidly degraded (16), losing its tumor-suppressive functions. On the opposite, in immune-excluded tumors, EMILIN-1 might promote immune evasion through alternative mechanisms.

Lastly, EMILIN-1’s role may shift during tumor evolution. During early tumorigenesis, EMILIN-1 may suppress transformation and proliferation. However, as tumors progress and the microenvironment becomes more complex, EMILIN-1 produced by recruited stromal cells might facilitate immune evasion or metastatic niche formation.

This framework generates several testable hypotheses: (i) Does EMILIN-1 produced by different cell types have distinct functional consequences? (ii) Do cleaved EMILIN-1 fragments antagonize full-length protein function? (iii) Does integrin expression pattern predict EMILIN-1’s functional role in a given tumor? (iv) Can spatial mapping of EMILIN-1 within tumors (perivascular vs. tumor core vs. invasive edge) explain functional heterogeneity? Addressing these questions will be essential to reconcile contradictory findings and harness EMILIN-1 biology for therapeutic benefit.

## 5 EMILIN-1 in CNS metastasis

In addition to primary tumors of the nervous system, Emilin-1 was recently reported to be involved in the progression of brain metastasis (BrM) (20). In the BrM microenvironment, EMILIN-1 is predominantly deposited by tumor-educated astrocytes rather than by the metastatic cancer cells themselves. It was shown that breast cancer BrMs can communicate with brain astrocytes and change their phenotype to engage astrocytes in tumor-promoting behavior. This resulted in an increased deposition of Emilin-1 by BrM-educated astrocytes, as documented by MS analysis of astrocytes` conditioned media and immunofluorescence analysis of mouse BrMs. In proximity with tumor cells, deposition of Emilin-1 led to a prominent increase in cyclin-dependent kinase 5 (Cdk5) levels by cancer cells, which in turn resulted in a downregulation of class I major histocompatibility complex (MHC-I) on the surface of BrMs (20), eventually leading to immune evasion and augmented immune camouflage (57). The pharmacological inhibition of Cdk5 with roscovitine (RSV) restored MHC-I expression in BrM-seeking cancer cells, enhancing their susceptibility to CD8+ T-cell-mediated killing, both *in vitro* and *in vivo*. Thus, RSV treatment reduced BrM outgrowth in preclinical models, which correlated with increased infiltration of CD8+ T cells and elevated expression of cytotoxic markers (granzyme B and perforin) in metastatic lesions. Furthermore, the RSV plus anti-PD-1 combination therapy significantly reduced BrM burden compared to monotherapies, accompanied by enhanced CD8+ T-cell infiltration and granzyme B expression (20). These observations strongly suggest that the Emilin-1–Cdk5 axis suppresses MHC-I-dependent antigen presentation, enabling immune escape, and its inhibition may overcome resistance to immune checkpoint blockade in BrM.

Notably, cancer cells treated with conditioned media from tumor-educated astrocytes with depleted *Emilin-1* could not effectively induce Cdk5 as compared to controls, whereas depletion of Emilin-1-producing astrocytes from the tumor microenvironment led to a stronger T-cell infiltration and immune response in BrMs. Interestingly, recombinant EMILIN-1 could directly induce higher Cdk5 levels in time- and dose-dependent manner when administered to breast cancer BrM-seeking cells *in vitro* (20), yet it is not clear whether this effect was due to increased mRNA expression of the Cdk5 gene or improved stability of Cdk5 protein. While the expression of EMILIN-1 in the healthy brain is rather low (primarily restricted to astrocytes, microglia, and endothelial cells), the microenvironment of astrocyte-infiltrated BrMs displays large amounts of deposited EMILIN-1 (20). Therefore, these findings suggest that EMILIN-1 may be a critical component of the communication between astrocytes and BrMs, establishing an immunosuppressive microenvironment and limiting the antitumor T-cell response. The mechanism by which Emilin-1 activates Cdk5 likely involves integrin-mediated signaling. EMILIN-1 binding to α4β1 or α9β1 integrins on GBM cells may trigger intracellular signaling cascades that activate Cdk5, though the detailed molecular pathway remains to be elucidated.

It is important to note that these findings are derived primarily from a single study using specific mouse models and breast cancer cell lines. The generalizability of these mechanisms to other brain metastasis types (e.g., lung cancer, melanoma) and their relevance to human disease require independent validation. Alternative explanations for the observed immune evasion phenotype (such as parallel pathways independent of EMILIN-1) cannot be excluded.

To date, this is the only comprehensive study examining EMILIN-1’s role in brain metastasis. The paucity of data in this area represents a significant knowledge gap, particularly regarding EMILIN-1’s potential involvement in brain metastases from other primary tumor types.

## 6 EMILIN-1 in other types of cancer

Multiple studies investigated the involvement of EMILIN-1 in cancers beyond the CNS, where its function was mostly linked to tumor vasculature changes. As discussed above (see 3.1.), EMILIN-1 primarily influences vasculature, whether in blood vessels or lymphatic vessels (29). Angiogenesis is essential for the formation of new blood vessels and development throughout the body (58); however, most solid tumors can stimulate angiogenesis to increase the supply of nutrients and oxygen (1), thereby facilitating malignant growth and metastasis. Multiple studies investigated EMILIN-1 function in the context of vasculature remodeling in extracranial tumors.

The disproportionate mechanistic detail provided for different tumor types reflects the uneven depth of investigation in the available literature. For brain metastasis, mechanistic studies remain relatively limited, whereas other metastatic sites have been more extensively characterized. We have therefore tailored our mechanistic descriptions accordingly, providing more detail where evidence permits and focusing on descriptive summaries where studies are sparse.

### 6.1 Gastric cancer

Capuano et al. demonstrated that Emilin-1 deficiency exacerbates gastric cancer development in multiple mouse models. Transgenic mice lacking functional EMILIN-1 displayed significant gastric mucosa thickening accompanied by more pronounced inflammatory infiltrate. In a model of chemically induced stomach carcinogenesis, the formation of gastrointestinal intraepithelial neoplasia and adenomas was markedly increased in *Emilin-1* mutant mice, with homozygous mutants more severely affected than heterozygous counterparts. Tumor analysis revealed increased lymphangiogenesis, suggesting that lymphatic vessels serve as a preferential route for tumor cell dissemination (59). Capuano et al. (2024) further showed that Emilin-1 deficiency exacerbates inflammation-driven colorectal carcinogenesis in mouse models, reinforcing its tumor-suppressive role throughout the gastrointestinal tract (59).

Qi et al. identified a synergistic relationship between EMILIN-1 and Tetraspanin 9 (TSPAN9) in gastric cancer using human gastric adenocarcinoma cell lines (SGC7901 and AGS). TCGA analysis revealed strong positive co-expression of EMILIN-1 and TSPAN9, with both proteins colocalizing in cancer cells and associating within protein complexes (60). While EMILIN-1 overexpression alone had no significant effect on tumor migration and invasion, TSPAN9 alone exerted tumor-suppressive effects. Notably, co-upregulation of both proteins produced a greater inhibitory effect than TSPAN9 alone, indicating functional synergy. Mechanistically, this synergy involves TSPAN9-mediated inhibition of the FAK-RAS-ERK1/2 pathway, potentiated by EMILIN-1 binding to α4β1 integrin via its gC1q domain. Additionally, EMILIN-1 co-localization stabilized TSPAN9 protein levels, delaying its degradation and prolonging its tumor-suppressive activity (60). While these findings in human gastric cancer cell lines and tissues provide important mechanistic insights, prospective clinical studies are needed to establish whether EMILIN-1 and TSPAN9 co-expression correlates with patient survival or therapeutic response in human gastric cancer cohorts.

The mechanisms by which EMILIN-1 suppresses gastric tumorigenesis likely involve multiple pathways: TGF-β inhibition, integrin-mediated anti-proliferative signaling via α4β1 and α9β1 integrins, stabilization of tumor suppressor proteins such as TSPAN9, and modulation of the inflammatory tumor microenvironment. These same pathways are frequently dysregulated in gliomas and other brain tumors, suggesting that EMILIN-1 may exert analogous tumor-suppressive functions in the CNS - an area warranting further investigation.

### 6.2 Skin cancer

The structural and regulatory functions of the extracellular matrix (ECM) are particularly pronounced in tissues with high ECM density, such as the skin (61), where it actively modulates cell proliferation (62). While common ECM proteins like tenascin C (63) and fibronectin often facilitate tumor growth, EMILIN-1 represents a distinct class of matrix components with potent tumor-suppressive properties (21). Mechanistic studies in chemically induced skin carcinogenesis model in *Emilin-1*−/− mice revealed a hyperproliferative microenvironment characterized by the activation of ERK1/2 and PI3K/Akt oncogenic pathways, coupled with a significant loss of the tumor suppressor PTEN (64).

This suppression of PTEN is driven by a sophisticated cross-talk between α4/α9 integrins and TGF-β signaling (17). In a physiological EMILIN-1-rich environment, integrin-mediated signals modulate Smad2 phosphorylation (at Ser245/250/255 residues), effectively dampening pro-tumorigenic TGF-β activity. However, the loss of EMILIN-1 disrupts this balance, leading to ERK-mediated downregulation of TGF-β levels and subsequent PTEN depletion (17, 21). Such mechanisms are of particular interest in the brain tumor microenvironment, where the PTEN/PI3K/Akt axis and TGF-β bioavailability are primary determinants of malignancy and immune evasion.

Furthermore, Emilin-1 acts as a critical gatekeeper of the vascular-lymphatic interface. Its absence triggers aberrant lymphangiogenesis, evidenced by an increase in LYVE-1+ vessels and altered lymph node morphology (65). This remodeling of the pre-metastatic niche significantly enhances the efficiency of tumor cell invasion, increasing metastatic rates from 25% to 75% in deficient models (21). Unlike other family members such as MMRN2, which appear redundant in this specific regulatory context, Emilin-1 uniquely restricts the formation of a pro-metastatic microenvironment (21, 65). Collectively, these preclinical findings suggest that Emilin-1 may be a critical regulator of tumor lymphangiogenesis and metastasis in this model system, highlighting a pathway that requires follow-up investigation in human cancers (**Table 1**). Given the recent discovery of meningeal lymphatics and their role in neuro-oncology, these findings suggest that EMILIN-1 may similarly govern the balance between tumor containment and lymphatic-mediated immune escape in the central nervous system (66).

**Table 1:** Clinical and translational landscape of EMILIN-1 in nervous system tumors.

Tumor type	EMILIN-1 expression	Primary cellular source	Evidence type	Association with prognosis	Key functional roles	Potential intervention points	Evidence strength	References
GBM	Downregulated in invasive tumor cell lines; absent from ECM	Tumor cells (Limited data)	Proteomics, cell line studies	Inverse correlation with invasiveness (cell lines studies)	Potential tumor suppressor; mechanism unclear	Restoration of EMILIN-1 expression; TGF-β pathway modulation	Moderate (limited functional validation)	(46, 47)
LGG	Elevated expression	Not specified	Transcriptomics, correlative analyses	High expression → poor survival	Correlates with immune infiltration; mechanism unvalidated	Immune checkpoint blockade; EMILIN-1 neutralization	Low (correlative only)	(48)
MB	Elevated in ECM	Not specified	Proteomics	Overexpressed in the SHH molecular subtype with worst prognosis	Unknown	ECM targeting	Limited	(46)
NB	Elevated expression, identified in disulfidptosis signature	Tumor cells	Bioinformatics, *in vitro* knockdown	Elevated expression associated with higher hazard ratios	Associated with disulfidptosis; knockdown reduces invasion/migration *in vitro*	Targeting disulfidptosis pathways	Low (mechanistic link unclear)	(53)
BrM (breast cancer)	Elevated (astrocyte-derived)	Tumor-educated astrocytes	Functional studies, mouse models	Promotes metastatic outgrowth	Activates Cdk5 → suppresses MHC-I → immune evasion	EMILIN-1 neutralization; Cdk5 inhibition; integrin blockade; combination with immunotherapy	Moderate (preclinical evidence; human validation limited)	(20)

### 6.3 Melanoma

The pre-metastatic niche (PMN) concept describes microenvironmental changes at distant sites that precede and facilitate metastatic colonization (67, 68). Primary tumors remodel local and systemic environments through vascular disruption, stromal cell activation, and ECM remodeling, while also co-opting immune cells to establish a positive feedback loop that supports PMN formation (67). Small extracellular vesicles (sEVs) secreted by primary tumors play key roles in this process by delivering DNA, RNA, and proteins to recipient cells, increasing vascular permeability via ZO-1 degradation, and recruiting fibroblasts, endothelial cells, macrophages, and bone marrow-derived cells to future metastatic sites (68–70).

López et al. investigated Emilin-1’s role in melanoma lymph node metastasis using three C57BL/6J and FVB mouse melanoma models: low-metastatic B16-F1, high-metastatic B16-F10, and lymph node-metastatic B16-F1R2 (56). Proteomic analysis of sEVs isolated from conditioned media revealed that Emilin-1 was among the most highly upregulated proteins in sEVs from the lymph node-seeking model compared to parental lines. Notably, intracellular Emilin-1 protein was undetectable in B16-F1R2 cells despite preserved mRNA expression, suggesting that Emilin-1 is proteolyzed and rapidly secreted via sEVs. This selective secretion mechanism may represent a strategy to remove tumor suppressor molecules, thereby promoting melanoma progression and metastasis (56). However, the functional consequences of sEV-mediated Emilin-1 secretion on recipient cells in the lymph node microenvironment remain to be determined.

### 6.4 Ductal breast carcinoma

Danussi et al. demonstrated that *Emilin-1*-/- mice (CD1 and C57BL/6 strains) exhibited increased mammary tumor proliferation and lymph node dissemination (21). Specifically, tumor growth and metastasis of transplanted syngeneic tumors were accelerated in *Emilin-1*-/- mice, accompanied by enhanced lymphangiogenesis both within the primary tumor and in sentinel lymph nodes. These findings establish EMILIN-1 as a negative regulator of lymphangiogenesis and lymphatic metastasis in peripheral tumors.

Translating these observations to human disease, Rabajdova et al. analyzed EMILIN-1 expression in ductal breast carcinoma (DBC) patients (71). This study found significantly decreased *EMILIN-1* mRNA and protein levels in stage GII DBC tumor tissues compared to healthy controls, consistent with a tumor-suppressive role. However, *EMILIN-1* mRNA levels were elevated in blood samples from stage GII and GIII patients, possibly reflecting sEV-mediated secretion similar to melanoma (56). Importantly, Rabajdova et al. found no correlation between *EMILIN-1* expression and disease stage, limiting its utility as a prognostic biomarker. Crucially, no correlation was found between *EMILIN-1* expression and disease stage, which fundamentally limits its utility as a prognostic biomarker. Several important caveats must be considered when interpreting these findings: the study is limited by its small sample size (n = 30 patients), the absence of functional validation, and the lack of mechanistic explanation for the observed discrepancy between tissue and blood EMILIN-1 levels. The observed discrepancy between tissue and blood EMILIN-1 levels remains poorly understood and requires confirmation in larger, independent cohorts, as well as mechanistic studies to determine its biological and clinical significance.

### 6.5 Colorectal cancer

Limited evidence exists regarding the potential involvement of EMILIN-1 in colorectal cancer. Capuano et al. (2024) also showed that Emilin-1 deficiency exacerbates inflammation-driven colorectal carcinogenesis in mouse models, supporting its tumor-suppressive role in the gastrointestinal tract (59). One study elucidated the role of Emilin-1 in migration and invasion of chemically induced colorectal cancer cells derived from *Ptp4a3*-/- mice, a model in which tumor cells exhibit impaired tumor migration, adherence, and shape compared to PTP4A3fl/fl control cells (72). Whole-body knockout of *Ptp4a3*-/- in mice led to the upregulation of several genes, including *Emilin-1*. This was further validated *in vitro* by treating colorectal tumor cells with a PTP4A3 inhibitor JMS-053, demonstrating an increased expression of several ECM proteins, including EMILIN-1. Therefore, PTP4A3 reduces migration and invasion of cancer cells at least partially through regulating EMILIN-1 expression.

### 6.6 Sarcomas and ovarian cancer

Maiorani et al. discovered that neutrophil elastase (NE) can proteolytically cleave EMILIN-1 into 3–4 fragments, abrogating its anti-proliferative activity (16). In neutrophil-infiltrated sarcomas and ovarian cancers, this neutrophil-mediated EMILIN-1 degradation impairs its tumor-suppressive functions. This mechanism may be broadly relevant across tumor types, including brain tumors with significant neutrophil or myeloid-derived suppressor cell (MDSC) infiltration. Neutrophil-mediated ECM remodeling could represent a general strategy by which tumors evade EMILIN-1’s growth-inhibitory effects. Since TGF-β may act as a major positive regulator of cancer cell G/M transition (34), EMILIN-1-mediated inhibition of TGF-β can be beneficial to control the proliferation of cancer cells.

## 7 Conclusions

The TME is a dynamic and complex network in which ECM components, such as EMILIN-1, play crucial roles in both physiological and pathological processes, including cancer progression and metastasis. EMILIN-1, a glycoprotein predominantly involved in the regulation of vascular and connective tissue homeostasis, exhibits context-dependent functions in development and cancer, especially in neural tissues (**Table 1**).

In primary nervous system tumors, EMILIN-1 demonstrates dual and often opposing roles (**Figure 2**). While its elevated expression correlates with poor prognosis in LGG and NB, promoting immune infiltration and disulfidptosis, its expression in GBM inversely correlates with tumor invasiveness. Notably, EMILIN-1 is absent in the GBM’s ECM but can be detected in MB’s ECM, suggesting the possible existence of tumor-specific regulatory mechanisms. As detailed in our proposed mechanistic framework, these divergent roles likely reflect differences in cellular source, protein processing, receptor availability, microenvironmental context, and temporal dynamics. These findings underscore EMILIN-1’s potential as a prognostic biomarker and therapeutic target; however, further research is needed to clarify molecular mechanisms underlying its effects on tumors.

**Figure 3:**
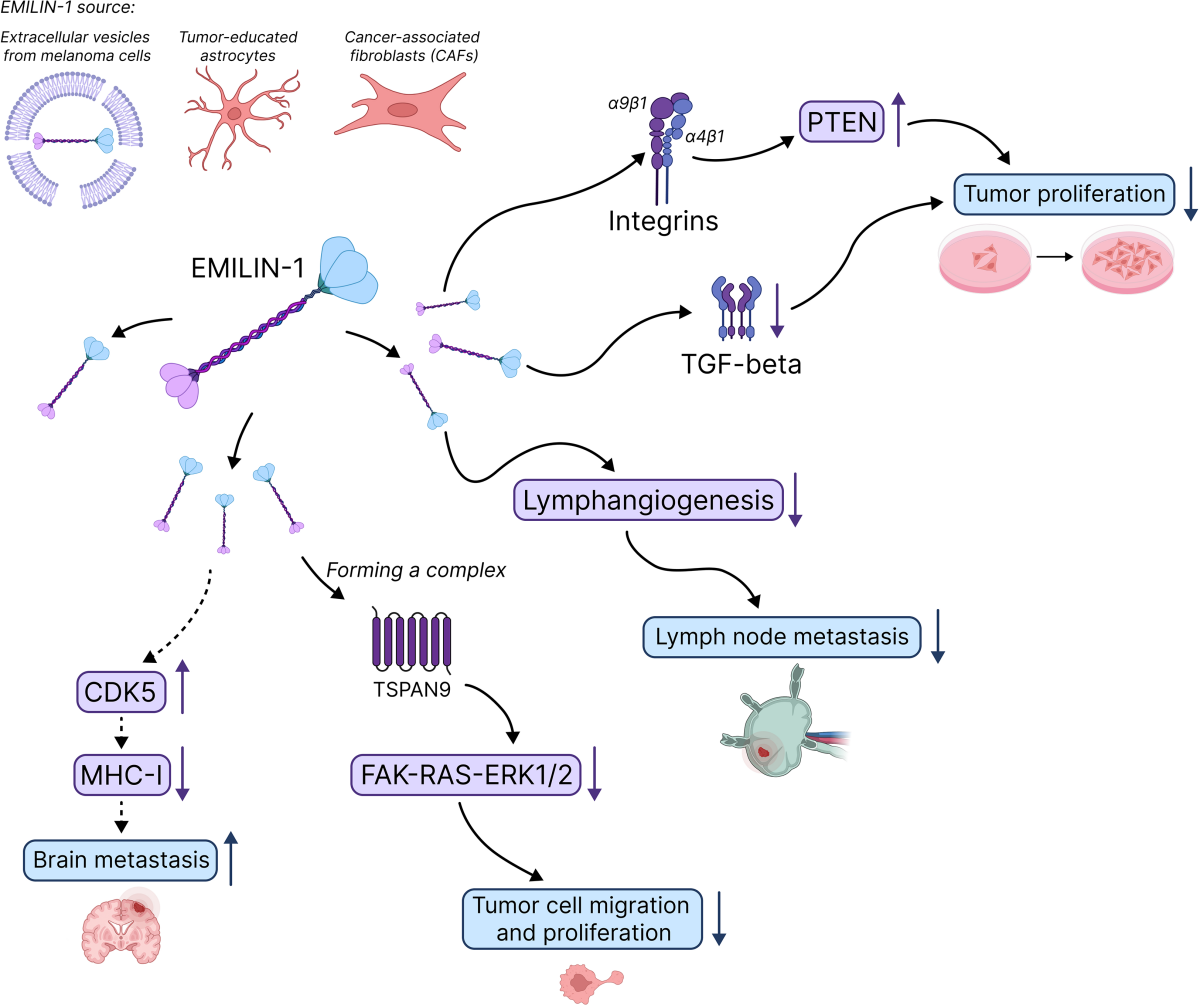
Pathophysiological mechanisms defining the function of EMILIN-1 in tumor development and progression. In cancer, EMILIN-1 often functions as a tumor suppressor by modulating signaling pathways such as TGF-β and integrin-mediated signaling. Its downregulation or proteolytic inactivation has been associated with increased tumor growth, angiogenesis, and metastasis in various cancers. However, EMILIN-1 can promote brain metastasis due to suppressing MHC-I expression on the surface of cancer cells, thus leading to immune evasion. Solid lines indicate established mechanisms; dashed lines indicate proposed or correlative relationships. Arrows denote associations or correlations rather than proven causal relationships unless specifically validated by functional studies.

In the context of BrMs, EMILIN-1 facilitates immune evasion by downregulating MHC-I expression in cancer cells through activating Cdk5 (**Figure 3**). Tumor-educated astrocytes deposit EMILIN-1 into the TME, thereby creating an immunosuppressive niche that hinders T-cell-mediated antitumor responses. Consequently, the targeting of EMILIN-1 or its downstream signaling pathways could emerge as a novel strategy to enhance immune surveillance in BrM. It is important to note, however, that the role of EMILIN-1 in brain metastasis is currently supported by limited experimental evidence. Additional studies examining diverse metastatic models, patient-derived samples, and alternative experimental systems are needed to confirm these findings and rule out model-specific artifacts. Therapeutic targeting of EMILIN-1 must consider potential risks including: (1) disruption of vascular homeostasis and blood pressure regulation, (2) unintended promotion of tumor growth in contexts where EMILIN-1 functions as a tumor suppressor, (3) impairment of lymphatic function, and (4) context-dependent effects that may vary by tumor type and stage. Tissue-specific or conditional targeting strategies may be necessary to minimize off-target effects.

Beyond the CNS, EMILIN-1 has been observed to exhibit tumor-suppressive properties in a range of cancers, including gastric cancer, skin cancer, melanoma, colorectal cancer and ductal breast carcinoma. In these tumor types, the loss or degradation of EMILIN-1 has been shown to correlate with increased proliferation, lymphangiogenesis, and metastasis (**Figure 3**). However, no clinical or interventional studies in humans have been conducted to date to confirm these mechanisms or establish a causal therapeutic role. Correlative analyses in human tumor samples are also lacking for most cancer types.

In summary, EMILIN-1 is a multifaceted ECM protein with diverse roles in cancer progression, immune regulation, and metastasis. The dual role of this protein, functioning both as a tumor suppressor and a promoter of metastasis, emphasises the significance of conducting context-specific investigations. Understanding the mechanisms behind EMILIN-1 depletion in tumors (e.g., gene deletion, transcriptional repression, or impaired sEV-mediated secretion), could provide valuable insights into potential therapeutic targets. Further investigation into the regulation of EMILIN-1 is crucial to uncover its therapeutic potential and validating its utility as a diagnostic and prognostic biomarker across cancer types. .
